# Exploring perceptions of dignity among older adults living in nursing homes: a qualitative study

**DOI:** 10.3389/fpsyt.2025.1616114

**Published:** 2025-07-02

**Authors:** Kejimu Sunzi, Hui Luo, Lina Yin, Yadi Li, Xin Zhou, Cheng Lei

**Affiliations:** ^1^ Department of Nursing, Deyang People's Hospital, Deyang, Sichuan, China; ^2^ Department of Nursing, Sichuan Nursing Vocational College, Deyang, Sichuan, China; ^3^ Department of Full Life Cycle Health Management, Deyang People’s Hospital, Deyang, Sichuan, China; ^4^ Department of Traditional Chinese Medicine (TCM), Deyang People's Hospital, Deyang, Sichuan, China; ^5^ Psychosomatic Medicine Department, Deyang People's Hospital, Sichuan, Deyang, Sichuan, China; ^6^ Department of Thoracic Surgery, Sichuan Clinical Research Center for Cancer, Sichuan Cancer Hospital and Institute, Sichuan Cancer Center, University of Electronic Science and Technology of China, Chengdu, China

**Keywords:** older adults, nursing homes, dignity, self-respect, qualitative

## Abstract

**Background:**

Rapid global population ageing has significantly increased the number of older adults requiring institutional care. In nursing homes, older residents frequently experience reduced autonomy, diminished social status, and restricted opportunities for meaningful social engagement, all of which can severely threaten their sense of dignity. Although dignity is widely acknowledged as a fundamental human right and is critical for maintaining older adults’ psychological well-being and overall quality of life, limited attention has been paid to understanding how Chinese nursing-home residents perceive, experience, and preserve their dignity. Clarifying these dignity-related experiences is essential to inform interventions and policies aimed at improving care practices and enhancing residents’ quality of life.

**Aim:**

To address the limited qualitative evidence on dignity in Chinese nursing homes, this study explored residents’ perceptions of dignity, the factors that undermine or enhance it, and the strategies they employ to preserve it.

**Methods:**

We adopted a descriptive phenomenological design to explore nursing-home residents’ perspectives and lived experiences regarding dignity. Between June and December 2023, we conducted semi-structured interviews with 35 nursing-home residents in western China (aged 65–92 years; length of stay 1 to > 7 years). Purposive maximum-variation sampling captured diversity in age, gender, functional status, and socioeconomic background. All interviews were audio-recorded, transcribed verbatim, and thematically analyzed using Colaizzi’s phenomenological procedure. Reporting adhered to SRQR guidelines.

**Results:**

The following four themes were identified: Older people’s perception of dignity, The influence of dignity on the older people, Factors affecting the promotion of dignity, Dignity maintenance strategies for the older people.

**Conclusion:**

Dignity in nursing-home settings is deeply influenced by physical dependence, the quality of staff–resident interactions, and the availability of meaningful social engagement. When dignity is preserved, residents display better psychosocial adjustment; when violated, they experience significant emotional distress.

**Recommendations:**

Nursing homes should implement staff training in person-centered, respect-focused care; design routines that maximize privacy and autonomy; and expand social and recreational programs. Future studies should develop and test targeted dignity-enhancement interventions and include family perspectives to create holistic, dignity-oriented care models.

## 1 Introduction

Population ageing is a well-documented global trend (1), presenting diverse challenges and societal responses across different regions. In 2019, an estimated 703 million people were aged 65 years or older, and this number is expected to double by 2050—meaning roughly one person in six will be over 65 (2, 3). The World Health Organization designates a society as “ageing” when ≥ 7% of its population is ≥ 65 years (4). Driven by longer life expectancy and lower fertility, this demographic shift has produced an unprecedented expansion of the older population globally (5); in many developed nations, for instance, average life span has almost doubled over the past two centuries (6, 7). United Nations projections indicate that older adults will comprise about 16% of the world’s population by 2050 (8), underscoring the worldwide significance of understanding and addressing the needs of this cohort.

With increasing longevity, a growing number of seniors worldwide experience limitations in self-care, and many eventually need institutional support—particularly nursing-home care, the provision and experience of which can vary significantly across different healthcare systems and cultural settings (9). Canadian projections, for example, suggest that long-term-care bed capacity may need to rise from 283,000 to 473,000 by 2045 (10), while on a global scale, more than three million older adults were already living in nursing homes in 2014 (11). Although these facilities provide essential safety and medical oversight, a common theme emerging from international research is that residents consistently report a desire to maintain lives that feel normal, meaningful, and as unconstrained as possible (12).

Ensuring overall health within nursing homes is therefore a crucial global concern, especially for residents who may lack consistent family support (13, 14). The World Health Organization defines health as “a state of complete physical, mental, and social well-being” (15, 16), and these dimensions are closely linked to older adults’ quality of life across diverse cultural contexts (17). Among these psychosocial dimensions, dignity holds particular importance, a concept recognized internationally: it encompasses self-worth, identity, and autonomy, as well as the respect accorded by others (2, 18). Preserving dignity in institutional care is critical—not only for mental health and quality of life, but also for protecting human rights, countering age-based stigma, and promoting social justice. Dignity is enshrined in Article one of the Universal Declaration of Human Rights and features prominently in international elder-care guidelines (19), reflecting its universal value. Empirical studies from various settings associate dignity with autonomy, independence, social inclusion, and attentive nursing care (20, 21). Nordenfelt further distinguishes four facets of dignity—human dignity, merit, moral stature, and identity—each of which is relevant to everyday interactions in nursing homes, though their salience and expression may be shaped by local cultures (22, 23).

A notable gap within the international literature concerns the understanding of cultural diversity in relation to dignity. Although dignity is recognized as a universal human value, its expression, the factors that compromise or support it, and the strategies employed for its maintenance can be culturally contingent. Consequently, a substantial body of in-depth qualitative research on dignity in nursing homes has predominantly originated from Western cultural contexts, notably the Nordic countries (21, 24).

Beyond mainland China, research in other Chinese societies, such as Hong Kong, offers related insights, though contextual differences must be acknowledged. For example, a study by Ho examining dignity among palliative care patients in Hong Kong found that while Chochinov’s Dignity Model was broadly applicable, certain subthemes (death anxiety, generativity/legacy, resilience/fighting spirit) manifested differently in the Chinese context (25). Importantly, this study identified new emergent themes reflecting cultural influences, such as “enduring pain” (viewed as a virtue), “moral transcendence,” “spiritual surrender,” and “transgenerational unity” (emphasizing family connection). These findings underscore the cultural and familial dimensions in the construct of dignity and the need for culturally sensitive approaches. However, it is crucial to recognize that the term “Chinese context” is not monolithic. Hong Kong possesses a distinct historical trajectory and healthcare system compared to mainland China. Similarly, studies on Chinese immigrant experiences in Western countries are shaped by acculturation and minority status, while research focused on specific care settings like palliative care or home-based long-term care address different populations and circumstances than those typically found in mainland Chinese nursing homes. While these studies offer valuable related insights, they do not directly capture the unique day-to-day experiences of older adults residing in the rapidly expanding nursing home sector within mainland China.

Within the Chinese cultural context, notions of ageing and dignity are profoundly intertwined with the Confucian ideal of filial piety (xiao), presenting a distinct perspective in the broader global discourse on elder care. Traditionally, adult children are expected to provide direct, hands-on care for their parents. Consequently, placing an older relative in a nursing home can be interpreted by families—and indeed by the residents themselves—as a failure to fulfill filial duty. Such perceptions risk undermining residents’ dignity of identity and moral stature, potentially leading to feelings of abandonment or social disapproval. Concurrently, the collectivist emphasis on maintaining social harmony and “face” may render residents hesitant to voice dissatisfaction, even when institutional routines infringe upon their autonomy. These culturally specific tensions, which can shape the experience of dignity in ways that either diverge from or align with those reported in other international contexts, underscore the imperative for care practices that are attuned to local conceptions of dignity.

In China, the rapid growth of the institutionalized older population has prompted concerns about how dignity is experienced and safeguarded in nursing-home settings. Yet, while a growing body of international literature explores dignity in elder care across various cultures, qualitative evidence capturing residents’ own perspectives from within the specific socio-cultural landscape of China remains limited. To address this gap, the present study explores how older adults living in Chinese nursing homes perceive dignity, what factors threaten or enhance it, and which strategies they use to preserve it. By adopting a qualitative approach, we aim to generate insights that can inform dignity-centered caregiving practices, potentially offering valuable understanding for both local and international audiences interested in culturally sensitive elder care.

## 2 Methods

### 2.1 Study design and setting

We employed a qualitative descriptive phenomenological design to explore how older adults experience dignity in nursing-home settings in Sichuan Province, China. Fieldwork was conducted in five nursing homes purposefully selected to maximize organizational diversity:

- Home A&B–Urban public facilities (100–500 beds each, downtown Chengdu);
- Home C–Suburban private, for-profit facility (≈200 beds, 20 km from city center);
- Home D&E–Rural collective-run homes (80–120 beds each, serving neighbouring townships).

All five homes provided 24-hour nursing care, basic medical services, and communal activity rooms, but differed in bed capacity, ownership, staffing ratios, and availability of rehabilitation programs. This heterogeneity allowed us to capture a wide spectrum of resident experiences and organizational contexts.

The study followed the Standards for Reporting Qualitative Research (SRQR) checklist to ensure transparency and maintain an audit trail (26)(**Supplementary Material 1**). Ethical approval was obtained from the Deyang People’s Hospital Ethics Committee (24).

### 2.2 Participants

From June to December 2023 we used a two-step strategy—purposive sampling followed by snowball referrals—to recruit nursing-home residents in western China.

In the Purposive stage, in consultation with administrators, nurses, and resident representatives, we created an initial list of candidates who varied in age, gender, length of stay, functional status, and socioeconomic background. Each candidate received an information sheet and could contact the research nurse directly or through a staff intermediary. After a private briefing, written informed consent was obtained.

In the Snowball stage, at the close of every interview, participants were asked:

“Can you think of another resident whose situation or background differs from yours (e.g., different age group, mobility level, or length of residence) and who might be willing to share their views?”

If the nominee agreed, the research nurse—again with staff assistance—approached that person, provided the same information sheet, and repeated the consent and screening procedures. This referral process was repeated until no new demographic combinations were suggested and data saturation was reached.

**Figure 1:**
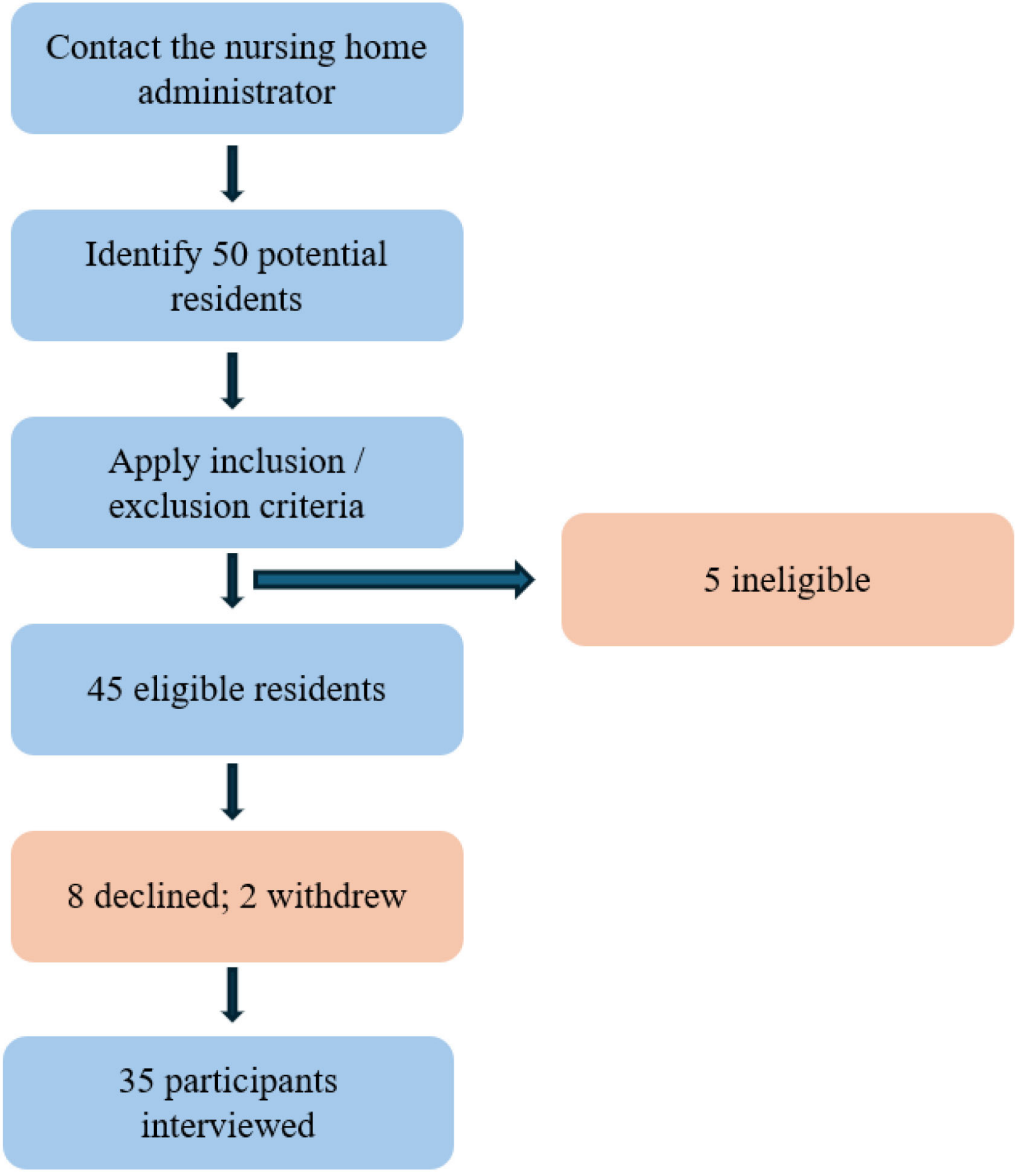
Flowchart of recruitment.

A total of 50 residents were approached. Eight declined, two withdrew part-way, and five failed to meet inclusion criteria, leaving 35 participants who completed interviews (**Figure 1**).

Participant selection criteria were as follows:

1. Individuals aged 65 years or older;

2. Able to communicate fluently in Mandarin Chinese (Putonghua), the official standard form of the Chinese language used in mainland China;

3. No psychosis or dementia, confirmed in three steps:

- recent chart review by the unit physician;
- Chinese MMSE cut-points (≥ 16 if no formal schooling; ≥ 20 with ≥ primary education) (27);
- a brief orientation conversation (time, place, own name) and the 6−item recall task conducted by the interviewer to ensure immediate and short−term memory adequacy;

4. Having resided in a nursing home for at least one year, ensuring participants had sufficient experience to provide rich descriptions of their lived experiences;

5. Capacity to give written consent and sustain a 30- to 60-minute dialogue.

Exclusion criteria included:

1. Terminal illness or acute medical condition requiring intensive treatment;
2. Ongoing psychotherapy or other active treatment likely to interfere with interview participation.

We continued recruitment and data collection until data saturation was achieved—that is, when additional interviews no longer generated new themes or insights. Data saturation was reached after conducting 30 interviews, and an additional 5 interviews were conducted to confirm saturation and ensure thorough representation of themes, justifying our final sample size of 35 participants.

### 2.3 Data collection

We developed a semi-structured interview guide (**Supplementary Material 2**) in three steps:

1. Scoping review of dignity-focused literature;
2. Multidisciplinary team discussions to translate key concepts into interview questions;
3. Pilot testing with two nursing-home residents, followed by refinements in wording and question order.

Interviews focused on residents’ day-to-day experiences and the challenges they encounter in maintaining dignity. All interviews were conducted between June and December 2023 in a quiet, private room provided by each facility (e.g., counselling office or unused activity room). The door was closed, a “Do Not Disturb” sign was posted, and only the interviewer and participant were present; residents could also request a different location if they felt more comfortable. Conversations lasted 30–60 minutes (mean≈45 minutes).

During each session the interviewer kept field notes, recording tone of voice, recurring descriptions, and personal reflexions to enrich later interpretation. Interviews were audio-recorded with written consent and transcribed verbatim within 48 h; files were anonymized and stored on a password-protected drive.

The research team—comprising specialists in nursing, psychology, and gerontology—used reflexive journals and regular debriefings to minimize bias. As a token of appreciation, participants received a supermarket voucher worth 200 CNY after the interview.

### 2.4 Data analysis

Data analysis commenced immediately following the transcription of the first interview and proceeded concurrently with data collection, guided by Colaizzi’s descriptive phenomenological method. Two independent coders (KS and CL), experienced in qualitative analysis, conducted the analysis collaboratively using NVivo 12™ software to enhance transparency and rigor.

All interview transcripts were imported into NVivo 12™ solely for data‐management and audit-trail purposes. Significant statements (Step 2) were highlighted and coded as “nodes” to facilitate retrieval; however, the formulation of meanings (Step 3) and clustering of themes (Step 4) were carried out through manual, line-by-line interpretation and reflexive discussion between the two coders. NVivo’s memo function was used to store our preliminary notes and reflexive observations, and its query tools were limited to checking coder agreement and tracking code frequency. No automated coding or text-mining features were employed, ensuring that phenomenological reflection—not software algorithms—guided each of Colaizzi’s seven steps. The resulting codebook, node hierarchy, and memos provided a transparent audit trail without compromising the descriptive phenomenological integrity of the analysis.

To support interpretation and deepen conceptual understanding, the analysis was informed by Nordenfelt’s theory of dignity, which conceptualizes dignity as comprising dignity of merit, moral stature, identity, and universal human worth. This framework provided a lens through which the researchers interpreted participants’ expressions of autonomy, self-worth, and interpersonal respect, allowing for a more nuanced understanding of how dignity is perceived and maintained in the nursing home context.

The analysis involved seven systematic steps as outlined by Colaizzi:

1. Familiarization:

Researchers thoroughly read and reread the transcripts multiple times to gain an in-depth understanding of participants’ experiences and contexts. Initial thoughts and reflections were documented as preliminary notes.

2. Identifying Significant Statements:

Researchers extracted statements directly relevant to the older adults’ experiences of dignity in nursing homes. Significant statements were carefully highlighted to reflect the core meaning of each participant’s experience.

3. Formulating Meanings:

Each significant statement was carefully reviewed to articulate its underlying meaning. Researchers maintained reflexivity through continuous discussions to ensure meanings accurately captured participants’ original expressions.

4. Clustering Themes:

Formulated meanings were organized into preliminary thematic clusters based on similarities. Researchers collaboratively reviewed these clusters, refining them iteratively until clear themes emerged, accurately reflecting the essence of participants’ experiences.

5. Developing an Exhaustive Description:

Themes were integrated into a comprehensive narrative description that encompassed the collective experiences of participants. The exhaustive description was continuously refined to ensure clarity, completeness, and fidelity to the original data.

6. Producing the Fundamental Structure:

The exhaustive description was condensed and synthesized into an essential structure representing the fundamental elements of participants’ experiences regarding dignity in nursing homes.

7. Seeking Verification of the Fundamental Structure:

To validate the accuracy and credibility of findings, the fundamental structure and key themes were shared with selected participants for feedback. Participants confirmed that the findings accurately represented their experiences. Feedback was then incorporated into the final analysis.

The analytical rigor was further strengthened through continuous comparative discussions, reflective journaling, and team consensus meetings. Additional details of the analytical process are provided in **Supplementary Material 3**.

### 2.5 Trustworthiness

We utilized various quality measures to ensure the credibility of our research findings: (1) Through purposive maximum variation sampling, participants were intentionally chosen to represent diverse categories of education level, socioeconomic status, and gender to generate a broad array of themes. (2) Audio recordings were transcribed into standardized text by a professional third-party service provider (https://www.iflyrec.com/). The transcribed texts were cross-checked against interview notes by two experienced researchers (KS and CL) to ensure the text’s accuracy and authenticity, preserving the “contextual restoration” as far as possible. (3) Two authors (KS and CL) independently analyzed the data, conducted consistency assessments of the formed codes, and resolved differences in interpretation through discussion, thus reducing the risk of imposing personal biases on the analysis. (4) To enhance the confirmability of research findings and the involvement of patients and the public, a preliminary report summarizing the research results and conclusions was sent to all 35 participants, a psychiatrist, and an expert in geropsychology to validate their reliability. (5) Since all participants conversed in Chinese, the accuracy of translated texts was verified by a bilingual researcher (HH, who obtained a Nursing master’s degree in China, a Ph.D. in the UK, and is proficient in qualitative research) after the final English versions of the theme descriptions were formed.

### 2.6 Ethics

This study strictly adhered to the Declaration of Helsinki and received ethical approval from the Ethics Committee of Deyang People’s Hospital (Approval No.: 2022-04-150-K01). All interviewees participated in this study voluntarily and could withdraw or revoke their data any time. All participants received an information flyer about the study and signed an informed consent form.

## 3 Results

### 3.1 Characteristics of participants

The participant group comprised 23 males and 12 females. Interviews were conducted between June and December 2023 (see **Table 1**).

**Table 1:** Demographic characteristics of participants (N=35).

Characteristic	Older adults
Sex	
Female	12
Male	23
Age (mean, SD)	68.75±2.89
Years in nursing home	
1-3years	10
4-6years	14
≥7years	11
Living status	
Alone	25
With others	10
Highest educational level	
High school diploma or less	16
College	12
Bachelor’s degree	4
Master’s degree	3

### 3.2 Themes and subthemes inducted from analysis

The main concern is the experience of dignity of the residents in institutions for the older people. Synthesis ultimately resulted in four major themes. Themes with essential exemplary quotations are presented below and in appendix.

#### 3.2.1 *Theme 1.* Older people’s perception of dignity

Everyone has their understanding and views on dignity, and there may be some differences in their sights, mainly including the subjectivity and relativity of dignity.

##### 3.2.1.1 Inner sense of self-worth

Older adults described dignity primarily as an inner, subjective sense of self-worth. Although scholars offer various formal definitions of dignity, participants emphasized that “real dignity is what I feel inside.”

“Everyone has their own dignity (Everyone, not only you young people, we old people also have our own dignity.” (Female, 75 years, living in nursing home for 3 years), one resident asserted.

For many, this inner dignity was closely tied to personal autonomy:

“I want conditions that permit me to go freely as it suits me, as long as I tell them. I feel that my life is worthy when I can decide for myself.” (Female, 70 years, living in nursing home for 5 years)

##### 3.2.1.2 Relational dignity

Older adults stressed that dignity is inherently relational—it depends not only on self-respect but equally on the respect shown by others.

“Dignity needs to be consolidated by yourself and respected by others before you feel dignified” (Male, 72 years, living in nursing home for 3 years), one resident explained.

Participants repeatedly described dignity as a mutual exchange of respect:

“If you respect others, they will respect you, and you will have dignity.” (Male, 68 years, living in nursing home for 4 years)

“This interplay of internal worth and external validation reflects what Nordenfelt terms dignity of merit and relational identity: individuals feel truly dignified when their intrinsic value is recognized within everyday interactions.” (Female, 70 years, living in nursing home for 5 years)

#### 3.2.2 *Theme 2.* The influence of dignity on the older people

After experiencing a loss of dignity, the older people may feel ashamed and have negative emotions and psychology. Good dignity, however, has a better effect on the older people.

##### 3.2.2.1 Psychosocial benefits of preserved dignity

Older adults emphasized that good dignity enables older persons to maintain a good mood, maintain better social connections and adapt to life in nursing homes more quickly.

“I’m not so rebellious, I don’t say: ‘oh no, what are they doing to me’. I think it’s awful if you start reasoning like that. Well, yes, I find it quite easy to adapt to a situation, at any rate. So then you feel a bit more relaxed and more well, we’ll see what happens and well, there’s just no choice, so that’s it.” (Female, 75 years, living in nursing home for 6 years)

##### 3.2.2.2 Psychological distress from dignity violation

Older adults reported that violations of their dignity triggered marked psychological distress, including feelings of humiliation, loneliness, anxiety, and a desire to escape institutional life.

“You see … it feels as if they treat us as if we don’t understand anything even though we have lived a whole life. Why doesn’t anyone speak to us? It is almost as if we are blockheads just because we are old … I suppose that is how it goes. There is nothing here for us; everything is supposed to be done quickly and fit into the right box. We are told when to eat, when to shower, everything. And no one ever says a word, no one says a word.” (Male, 74 years, living in nursing home for 7 years)

It can even cause some people to feel scared and lonely.

“They are all gone; all are already gone. Kith and kin, all of them. And I am the only one of them still running around. So it is. (…).” (Male, 73 years, living in nursing home for 5 years)

When the experience of dignity is not good, the older people may have the idea of running away from the nursing home, the desire to return to the original family, and even begin to self-doubt and deny their value. It takes them longer to adjust to life in a nursing home.

“It was terrible to leave (home) like that. For a long while, I couldn’t sleep. I could not sleep at night, but now I can. I’ve had to adapt.” (Female, 70 years, living in nursing home for 7 years)

“I want to live at home,” stressing tacitly that, for him, the nursing home was not a home but rather a place that had robbed him of his freedom. (Male, 75 years, living in nursing home for 6 years)

#### 3.2.3 *Theme 3.* Factors affecting the promotion of dignity

##### 3.2.3.1 Functional decline and bodily dependence

Functional decline and bodily dependence were repeatedly cited as potent threats to dignity. Participants explained that physical deterioration erodes self-image, privacy, and freedom of choice, forcing them into humiliating dependence on caregivers for the most intimate tasks.

“It’s horrible not being able to take care of myself. I can always get help but it’s horrible to wake up when you wet your own bed. Everything you do you’re dependent on others. For example, when you need to go to the toilet. It doesn’t feel good to ask for help going to the toilet, just like babies” (Female, 10 years, living in nursing home for 4 years), one resident said.

Another participant added,

“If your relative is with you, they talk to the relative and they say ‘well she has the odd back ache and etc., etc.’ and when they do that to me I say, ‘look, I am here, so talk to me.’… I find it insulting; they talk above you like you are a mute, deaf and dumb….” (Male, 71 years, living in nursing home for 4 years)

##### 3.2.3.2 Care staff interactions and care routines

The dignity of residents was closely tied to how staff interacted with them and managed daily care routines. Respectful, person-centered practices—knocking before entering, preserving privacy, offering reassurance—helped minimize embarrassment and reinforced residents’ sense of worth:

“They are nice here. I have never had problems contacting the staff. I just say I peed all over the floor.” (Female, 69 years, living in nursing home for 3 years)

“We’ll take care of it, don’t worry. We are here to help you. Just call when you need us.

Yes, they’ll never come in without knocking, they always ask how you are. And if you are looking particularly good on a particular day, there’s a nurse from Surinam and he’ll say:’What lovely eyes you have’.” (Male, 75 years, living in nursing home for 8 years)

“They keep you clean and look after you. Like I say, I never got looked after at home like I do here … I don’t think there are any [care-staff] in here who wouldn’t go out of their way to help me if I need it.” (Male, 71 years, living in nursing home for 9 years)

Conversely, staff shortages and task-focused routines could undermine dignity when timely assistance was not available:

“They don’t have enough nurses to get you up to go to the toilet, so they put these things on you … so you have to put up with something … it was hard … and I said ‘couldn’t we have a bedpan?’ Well,[chuckles uncomfortably] I’ve got them the odd time, but not very often. Well, now I’m used to it.” (Female, 69 years, living in nursing home for 5 years)

##### 3.2.3.3 Opportunities for social engagement

Opportunities for social engagement were identified as key to maintaining dignity, as limited social interaction and emotional support led to feelings of isolation, diminished self-worth, and a lack of meaning in life. Participants reported that opportunities for engagement in recreational activities significantly enhanced their dignity and social connections.

“I am very fond of dancing. I have danced for 20 years. Now I go dancing once a week. The bus come and picks me up. I am the only one from this place.” (Male, 70 years, living in nursing home for 10 years)

““I’ve been dancing for 20 years,” one resident said. “Now I go once a week. The bus picks me up, and I’m the only one from this place. I love it.”” (Male, 73 years, living in nursing home for 6 years)

“Meeting in the common room for chess and conversation makes me feel as if I’m with family again—being needed and heard lifts my whole day,” another participant added.” (Female, 70 years, living in nursing home for 5 years)

#### 3.2.4 *Theme 4.* Strategies to maintain dignity

As for how to maintain their dignity, the older people have come up with some of their own strategies.

##### 3.2.4.1 Cognitive re−appraisal and acceptance

Older adults described using cognitive re-appraisal and acceptance to protect their dignity: they deliberately reframed the nursing-home stay as an opportunity for social connection and meaningful activity rather than a loss of independence.

“My children say their mom joins everything—even dancing in a wheelchair” (Male, 78 years, living in nursing home for 8 years), one resident laughed, illustrating her decision to focus on what she can still enjoy.

Another explained, “My family said I had to move here, so I told myself it would be a fresh start.” (Female, 70 years, living in nursing home for 5 years)

“The staff stop to chat for ten or fifteen minutes—they’re not just changing sheets; they’re company.” (Female, 72 years, living in nursing home for 5 years), a participant noted, emphasizing how she actively interprets everyday interactions as friendship.

##### 3.2.4.2 Reasserting identity through valued roles

Older adults reasserted their identity by drawing on valued life roles, maintaining a positive self−image, and helping others—acts that restored confidence and dignity.

“My mother’s father was the mayor of a town. I was quite well born. My family was from the samurai class. So, in that rural area, my family was quite famous.” (Male, 70 years, living in nursing home for 6 years)

, one resident proudly recalled, anchoring her sense of worth in her family’s history.

Another emphasized appearance as a daily statement of self−respect, “I still try to look nice every day, how I look means everything. So I am very pleased that the nurses are interested in my appearance and help me.” (Male, 70 years, living in nursing home for 5 years)

A third participant found purpose in supporting peers, “I read letters aloud for those with poor eyesight—being useful makes me feel like myself again.” (Female, 77 years, living in nursing home for 9 years)

Through storytelling, self−presentation, and contributory roles, residents reclaimed a coherent identity and reinforced their dignity within the nursing−home setting.

##### 3.2.4.3 Planning for a dignified end of life

In nursing homes, death is an unavoidable topic, and how to approach it with dignity is a major concern for residents. Many participants first, described coming to terms with mortality:

“Eventually, as I get older, I’ll have to go there too. It is what it is. I don’t know whether I’ll live another 20 years or not. I think it’s natural for things to go like that. From here on, what is important is to try to live so that I don’t invite the hate of others.” (Male, 72 years, living in nursing home for 4 years)

Having accepted the inevitability of death, they then made practical preparations—often planning their own funerals to spare their families additional stress:

“I have already arranged the burial,…, so that they won’t have so much work with me … Everything is already written down, mass … flowers … a cremation and then it’s all done. One does not need to make a single additional call.” (Female, 70 years, living in nursing home for 7 years)

Finally, residents expressed a clear wish for a peaceful and dignified passing:

“I just want to let myself die with dignity, to be able to die in a dignified way as if I was asleep.” (Male, 78 years, living in nursing home for 9 years)

## 4 Discussion

We conducted a systematic exploration of the dignity experiences of older residents in nursing homes using qualitative research methods. Our study recruited older individuals living in nursing homes as participants, aiming to provide insights into the barriers and facilitators affecting their dignity. Additionally, we identified strategies to enhance the dignity of these older adults in nursing home settings. Our results underscore the profound importance of dignity for older adults, a finding that resonates across numerous studies in diverse cultural settings. The emphasis our participants placed on having a degree of autonomy to maintain dignity, and their view that passive acceptance often leads to lower social status, self-esteem, and confidence, aligns with international research highlighting autonomy as a cornerstone of dignity (28). This is particularly reflective of Nordenfelt’s concept of “dignity of identity,” which encompasses autonomy and self-respect as integral to an individual’s sense of self (29). Similarly, Jacobson, Oliver, and Koch also underscore autonomy as a central tenet in their conceptualizations of dignity in care (30). Future research should continue to build on these insights, potentially developing and testing evidence-based programs to reach consensus on culturally nuanced definitions of dignity and formulate practical guidelines to enhance the dignity experience for older adults. Our findings reveal that the attitudes and behaviors of institutional staff towards the older adults directly affect their perceived dignity. The dignity of the older population can be significantly compromised if they are insulted. Dignified care for the older adults cannot be achieved without ensuring dignified treatment of the staff in older care institutions. As a supplementary form of hospice care, dignified care provides comprehensive support for the body, mind, society, and spirit of older adults. It improves their sense of self-worth and life significance, allowing them to spend their later years with dignity. Dignified care not only addresses the physical needs of the older adults but also focuses on their psychological care and comfort. It embodies respect for their personality and meets their higher-level needs. A systematic review identified respectful and empathetic staff attitudes as key facilitators of dignity (31). Furthermore, Slettebø et al., in their study in a Western context, found that feeling disregarded or objectified by healthcare professionals was a primary threat to residents’ dignity (21). Our findings are also consistent with resources like which compile evidence-based practices emphasizing that respectful communication and positive interactions from staff significantly boost residents’ dignity. The International Council of Nurses (ICN, 2012) code states, “Inherent in nursing is respect for human rights, including cultural rights, the right to life and choice, to dignity, and to be treated with respect (32). Furthermore, the ICN identifies maintaining human dignity as an ethical goal of nursing. The comprehensive support for body, mind, society, and spirit provided by dignified care, which enhances self-worth and life significance, can be seen as aligning with Maslow’s hierarchy of needs, particularly esteem and self-actualization.

As the primary caregivers for older adults, nursing staff should first improve their skills and capabilities to ensure that the older adults receive high-quality care. They should maintain a polite attitude during the care process, show full respect to the elderly, and avoid any form of insult or discrimination. This is strongly supported by the qualitative evidence synthesis by Sunzi et al., which stresses that nursing home staff must prioritize residents’ dignity through respectful care and the preservation of independence (33). It is recommended to enhance training in geriatric nursing and humanistic care for new registered nurses to improve their ability to provide compassionate care (33). In their daily work, nurses should strengthen communication with older adults, listen attentively, understand their needs, provide timely assistance, and establish trusting and cooperative relationships with them.

Our study highlighted that social participation and interaction are vital for older residents, who often experience weakening social ties and increased dependency. The positive impact of social interactions on quality of life is well-documented. Our findings, indicating that participation in social activities improves older adults’ health and well-being, are supported by international research where social inclusion and meaningful engagement are central to fostering belonging and respect (34). The finding that negative social interactions can make residents feel worthless and lose autonomy, damaging their dignity, where older adults expressed fears of becoming a “nobody” and being disregarded (21). The need for social support in forming a new identity within the nursing home, and the role of activities like sports, singing, dancing, and painting in establishing good interpersonal relationships, is consistent with research showing that recreational activities build confidence and social connections, contributing to dignity (33, 34). Participation in social activities improves the health of older adults from multiple perspectives, ultimately resulting in lower mortality and higher survival rates (35). Both exercise and socializing have positive and negative effects on older adults. Negative social interactions can make them feel worthless and lose their autonomy, thus damaging their dignity. Therefore, older adults living in nursing homes need the help of social members to form a new identity within the nursing home environment. Good interpersonal relationships can be established and developed by participating in social activities such as sports, singing, dancing, and painting. Research reveals that physical activity can significantly reduce anxiety, stress, and depression, maintain mental health, and ensure psychological vitality; it also improves the overall quality of life (36). Participating in social activities not only exercises the body but also helps older adults find a sense of belonging and identity, giving them a sense of life’s meaning and promoting their experience of dignity. Research shows that successful aging requires maintaining high physical activity levels and continuous participation in social and productive activities (37). Previous studies indicate that physical activity helps reduce depression by boosting confidence and physical efficacy through bodily movement (38–40).

However, the manifestation and emphasis of dignity components can differ culturally. Our findings can be compared with specific themes from other Chinese contexts. For instance, a study in mainland China, identified “self-esteem (sense of self-worth and becoming a burden)” and “perceived value to society (being respected and belonging)” as central (41). The concern about “not becoming a burden” is particularly poignant in cultures deeply influenced by filial piety (xiao), where the traditional expectation is family-based care, and institutionalization might evoke feelings of guilt or inadequacy, impacting dignity. Similarly, Ho et al., adapting Chochinov’s Dignity Model for palliative care patients in Hong Kong, identified culturally resonant themes like “enduring pain” (as a virtue), “moral transcendence,” and “transgenerational unity” (emphasizing family connections) (25, 42). While our study may not be in palliative care, the emphasis on family and interconnectedness in Ho et al.’s findings offers a valuable comparative point regarding how dignity is constructed within a broader Chinese cultural sphere (25).

Our findings on the overall dignity of older people in nursing homes offer valuable insights for future research in this field. Establishing a consensus definition of dignity for older people in nursing homes is crucial, as greater conceptual clarity would lead to standardized measures and more targeted interventions.

### 4.1 Implications for nursing practice

Nursing Home Staff is one of the best professions for conveying human kindness and empathy to the older people. Our findings highlight the importance and potential benefits of involving family members in care, especially given their perceptions of older people (e.g., likes, dislikes, stressors), this is critical information for the caregiver. Family members can also be encouraged to provide social support to older persons (such as in-person care, frequent visits) and communication (such as talking about information in society and conveying daily messages from family members). Encourage children to visit the older people, so that the older people feel that they have not been abandoned, not lost contact with the original life.

### 4.2 Strengths and limitations

#### 4.2.1 Strengths

- This qualitative study provides a deep and nuanced understanding of how older adults in nursing homes in West China perceive and experience dignity. The use of qualitative methods allows for rich, detailed data that can capture the complexities of these experiences.
- It explores the real life experience of the older people in nursing homes.
- The findings offer practical recommendations for improving the care and dignity of older adults in nursing homes. These insights can guide caregivers, policymakers, and healthcare providers in implementing effective strategies.

#### 4.2.2 Limitations

- The study is conducted in West China, and the findings may not be generalizable to other regions or countries due to cultural and social differences. Further research in diverse settings is needed to validate the findings.
- The qualitative nature of the research means that the findings are based on subjective interpretations of the participants’ experiences. While this provides depth, it may also introduce bias.
- The study captures the experiences of older adults at a specific point in time. Longitudinal studies would be beneficial to understand how perceptions of dignity may change over time.

## 5 Conclusion

Our qualitative findings highlight that factors such as nursing‐staff attitudes, family support, and residents’ physical functioning all shape older adults’ dignity in nursing homes. However, family and caregiver perspectives on dignity‐promoting practices remain under‐explored, and future studies should engage these stakeholders to identify specific actions that uphold residents’ sense of worth. Moreover, subsequent research should establish measurable outcomes—for example, validated dignity scales, quality‐of‐life indices, and caregiver–resident satisfaction metrics—to rigorously evaluate the impact of dignity‐enhancement interventions. Methodological rigor can be further strengthened through systematic reflexivity reporting and interdisciplinary research teams that include clinicians, social scientists, and family‐care experts. Finally, developing a consensus definition of dignity for older adults will support the creation of standardized measures and ensure consistency across future intervention studies in nursing‐home settings.

## Data Availability

The original contributions presented in the study are included in the article/**Supplementary Material**. Further inquiries can be directed to the corresponding author.
