# Supplementary material for: Exploring perceptions of dignity among older adults living in nursing homes: a qualitative study
Source: Front Psychiatry. 2025 Jul 2;16:1616114. doi: 10.3389/fpsyt.2025.1616114 (PMC12264539; doi:10.3389/fpsyt.2025.1616114)
Supplement: Supplementary file 1 [file Table1.docx]

**Supplementary file 1**

**Interview schedule**

| Items | Interview questions |
| --- | --- |
| Before starting the interview | Introduce themselves |
|  | Hello, my name is Kejimu Sunzi, I would like to talk to you about some issues regarding dignity. I would like to ask you about your personal information such as name, age, position, and setting. You can stop or quit the interview process at any time. Do you agree to participate in this study? If you agree, please sign the informed consent form.   - Could you tell me about personal information? - How many years you live in nursing homes? |
| Topic guide | - How do you understand the word "dignity"? What does dignity mean to you? - In your daily life at the nursing home, what kinds of situations make you feel respected or that your dignity is maintained? - Have you ever experienced situations where you felt your dignity was violated? Could you share more about that experience? - How important is dignity to your overall quality of life in the nursing home? - How does feeling respected or having your dignity maintained affect your emotional and psychological well-being? - When you feel your dignity is upheld, does it change how you approach your daily activities or social interactions? - In what ways does a sense of dignity influence your self-identity and life satisfaction? - What factors do you believe contribute to maintaining or enhancing your sense of dignity in the nursing home? - Are there aspects of the nursing home's environment or policies that make you feel your dignity is protected? - Are there any factors that make you feel your dignity is threatened or not valued? - What actions do you think the nursing home staff can take to help you maintain your dignity? - What personal strategies do you use to maintain and enhance your sense of dignity in daily life? - What specific measures would you like the nursing home management to implement to improve your and other residents' sense of dignity? |
| The end | Do you have anything else to add?  Thank you for participating and providing valuable information about your experience in nursing homes. |
