# Supplementary material for: Exploring perceptions of dignity among older adults living in nursing homes: a qualitative study
Source: Front Psychiatry. 2025 Jul 2;16:1616114. doi: 10.3389/fpsyt.2025.1616114 (PMC12264539; doi:10.3389/fpsyt.2025.1616114)
Supplement: Supplementary file 2 [file Table2.docx]

| **Step** | **Description** |
| --- | --- |
| 1.Familiarisation | The researcher familiarises ourselves with the data, by reading through all the participants accounts several times |
| 2.Identifying significant statements | The researcher identifies all statements in the accounts that are of direct relevance to the phenomenon under investigation |
| 3.Formulating meanings | The researcher identifies meanings relevant to the phenomenon that arise from a careful consideration of the significant statements. The researcher must reflexively bracket” our pre-suppositions to stick closely to the phenomenon as experienced (though Colaizzi recognizes that complete bracketing is never possible). |
| 4. Clustering themes | The researcher clusters the identified meanings into themes that are common across all accounts. Again bracketing of pre-suppositions is crucial, especially to avoid any potential influence of existing theory |
| 5. Developing an exhaustive description | The researcher writes a full and inclusive description of the phenomenon, incorporating all the themes produced at step 4. |
| 6. Producing the fundamental structure | The researcher condenses the exhaustive description down to a short, dense statement that captures just those aspects deemed to be essential to the structure of the phenomenon. |
| 7. Seeking verification of the fundamental structure | The researcher returns the fundamental structure statement to all participants (or sometimes a sub-sample in larger studies) to ask whether it captures their experience. We may go back and modify earlier steps in the analysis in the light of this feedback. |
